# Isosteric substitution in cationic-amphiphilic polymers reveals an important role for hydrogen bonding in bacterial membrane interactions†

**DOI:** 10.1039/c6sc00615a

**Published:** 2016-04-07

**Authors:** D. S. S. M. Uppu, M. M. Konai, U. Baul, P. Singh, T. K. Siersma, S. Samaddar, S. Vemparala, L. W. Hamoen, C. Narayana, J. Haldar

**Affiliations:** a Chemical Biology and Medicinal Chemistry Laboratory, New Chemistry Unit, Jawaharlal Nehru Centre for Advanced Scientific Research (JNCASR) Jakkur Bangalore India-560064 jayanta@jncasr.ac.in; b The Institute of Mathematical Sciences (IMSc), C.I.T. Campus Taramani Chennai 600113 India; c Light Scattering Laboratory, Chemistry and Physics of Materials Unit, Jawaharlal Nehru Centre for Advanced Scientific Research (JNCASR) Jakkur Bangalore India-560064; d Bacterial Cell Biology, Swammerdam Institute for Life Sciences (SILS), University of Amsterdam Amsterdam 1098 XH The Netherlands

## Abstract

Biomimetic antibacterial polymers, the functional mimics of antimicrobial peptides (AMPs), targeting the bacterial cell membrane have been developed to combat the problem of antibiotic resistance. Amphiphilicity, a balance of cationic charge and hydrophobicity, in these polymers has been shown to be pivotal for their selective interactions with anionic lipid membranes of bacteria instead of zwitterionic mammalian (human erythrocyte) membranes. However, it is unclear if and to what extent hydrogen bonding in amphiphilic antibacterial polymers contributes to this membrane binding specificity. To address this, we employ isosteric substitution of ester with amide moieties that differ in their potency for hydrogen bonding in the side chains of *N*-alkyl maleimide based amphiphilic polymers. Our studies reveal that amide polymer (AC3P) is a potent antibacterial agent with high membrane-disrupting properties compared to its ester counterpart (EC3P). To understand these differences we performed bio-physical experiments and molecular dynamics (MD) simulations which showed strong interactions of AC3P including hydrogen bonding with lipid head groups of bacterial model lipid bilayers, that are absent in EC3P, make them selective for bacterial membranes. Mechanistic investigations of these polymers in bacteria revealed specific membrane disruptive activity leading to the delocalization of cell division related proteins. This unprecedented and unique concept provides an understanding of bacterial membrane interactions highlighting the role of hydrogen bonding. Thus, these findings will have significant implications in efficient design of potent membrane-active agents.

## Abstract 2

The important role of hydrogen bonding in the interactions of cationic-amphiphilic polymers with bacterial membranes has been reported.

## Graphical abstract

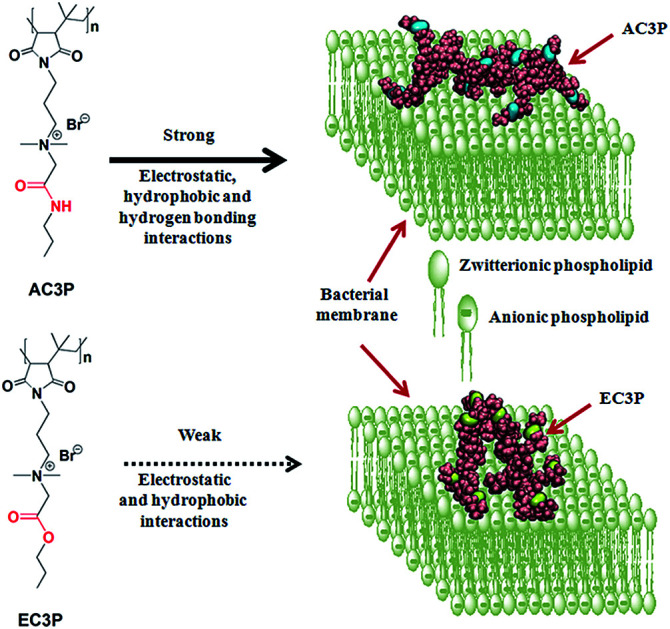

## Introduction

The alarming increase of resistant superbugs coupled with the diminishing antibiotic pipeline creates an urgent need for the development of new antimicrobial agents which exert novel mechanisms of action.^1,2^ In the past two decades, antimicrobial peptides (AMPs) that interact primarily with the microbial membrane have received increasing attention due to their ability to combat multi-drug-resistant microbes.^3–8^ Targeting the bacterial membrane indeed has been shown to possess low propensity for the development of bacterial resistance.^9^ However, the limited success of AMPs in clinical applications is due to their high toxicity (for *e.g.* hemolysis), low stability (susceptible to proteases) and more importantly, high manufacturing costs that prevent their large scale industrial production.^3–5^ More recently, β-peptides,^10^ peptoids,^11^ arylamide oligomers,^12^ oligo acyl lysines,^13^ γ-peptides,^14^ antimicrobial polypeptides^15,16^ and diverse polymeric AMP analogs^17–24^ have been developed to address the problems that limit AMPs from reaching the clinics. Like AMPs, these polymers possess broad spectrum of antibacterial activity but importantly unlike AMPs they can be prepared with low cost at large scale. The peptide backbone of AMPs is labile to the proteolytic degradation whereas the stable non-peptide backbone of most of these polymers makes them more suitable for *in vivo* use.

Amphiphilicity, a key structural feature of AMPs, has been optimized in antibacterial polymers reported so far. This amphiphilicity which is a fine balance of hydrophobicity and cationic charge has been shown to play a key role in the selective interaction of these polymers with the bacterial membranes instead of mammalian (*e.g.* human erythrocyte) membranes.^19^ Electrostatic interactions bind the cationic polymers to the bacterial membranes that contain negatively charged phospholipids whereas hydrophobic interactions facilitate the binding and insertion into the lipid bilayers. The selective interactions of amphiphilic polymers with bacterial membranes results from the fact that the eukaryotic cell membranes generally contain a higher proportion of zwitterionic phospholipids and are rigid and possess high membrane order due to the presence of cholesterol.^19^ Despite these differences, driving the selective interactions of amphiphilic polymers towards bacteria has remained elusive. An important and yet unexplored question is how hydrogen bonding contributes to the bacterial membrane specific interactions of amphiphilic polymers. In this study, we examined the role of hydrogen bonding in tuning the selective interactions with bacterial membranes using maleic anhydride based polymers.

**Fig. 1:**
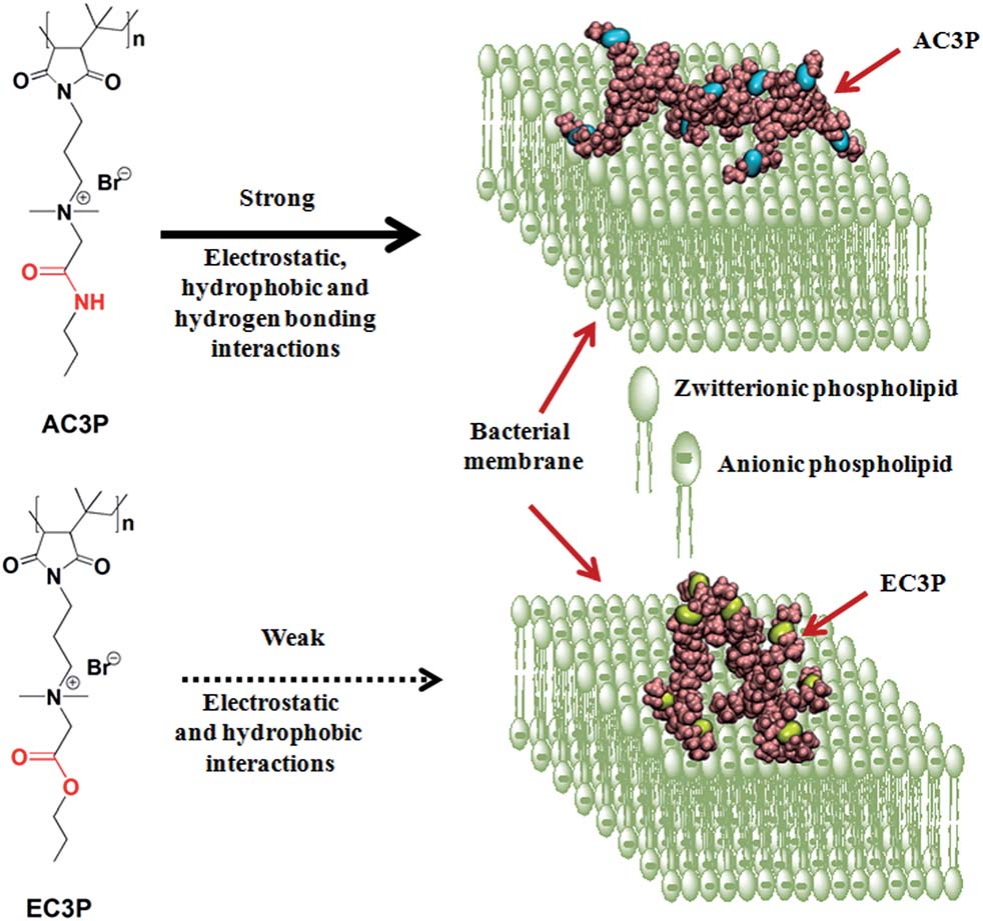
Schematic representation of the effect of isosteric substitution in cationic-amphiphilic polymers towards bacterial membrane interactions.

To investigate the role of hydrogen bonding, we exploited the concept of isosteric substitution of ester with amide moieties in the side chains of poly(isobutylene-*alt-N*-alkyl maleimide) based amphiphilic polymers prepared from their maleic anhydride precursor polymer. Substitution of an ester with an amide is isosteric and also varies in the potency of hydrogen bonding. We found that the amide polymers (AC3P) displayed high antibacterial activity compared to their ester (EC3P) counterparts. To probe these differences, we performed biophysical experiments and molecular dynamic (MD) simulations on bacterial model lipid bilayers. Collectively, our studies provided the evidence that AC3P, but not EC3P, form strong interactions including hydrogen bonding with the phosphate head groups of bacterial model lipid bilayers (Fig. 1). Studies performed to understand the mode of action revealed membrane-disruptive activity that led to the dissipation of membrane potential, pore formation, energy depletion and mis-localization of essential cell division proteins.

## Results and discussion

### Rational design

**Fig. 2:**
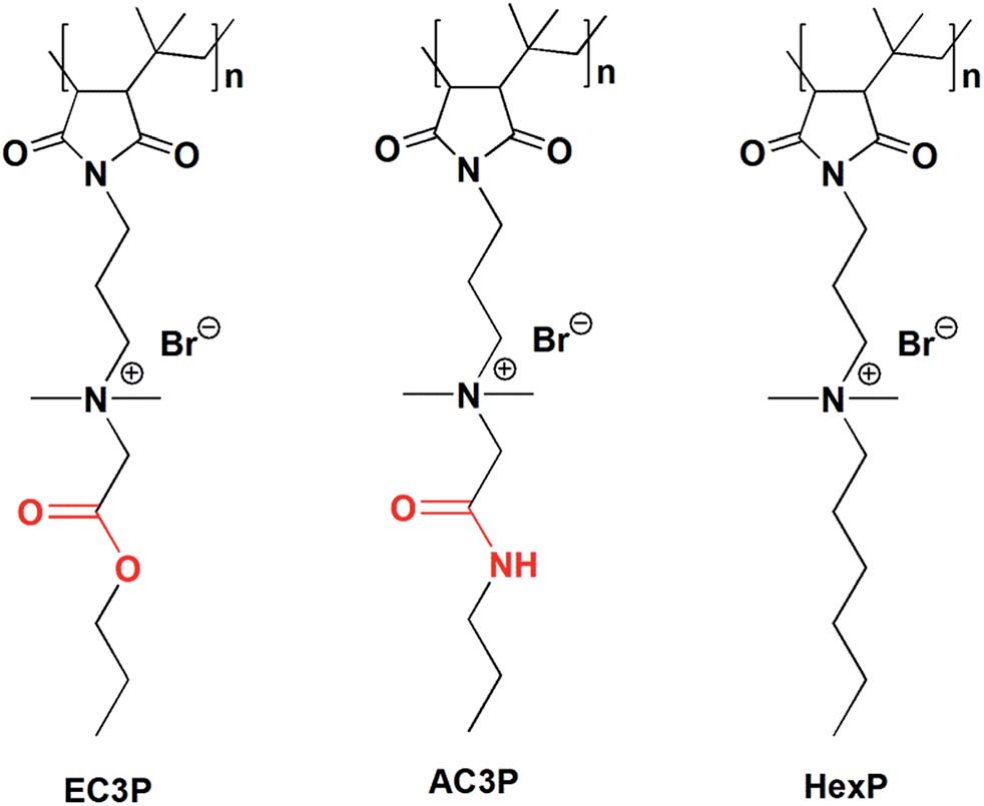
Chemical structures of cationic-amphiphilic polymers.

Amide and ester moieties, in general, possess differences in their ability to form hydrogen bonds. We chose this simple replacement of ester with amide moieties, called as isosteric replacement, in the side chains of poly(isobutylene-*alt-N*-alkyl maleimide) based amphiphilic polymers. An amide bond is isosteric to an ester because the –O– in the ester is of near-equal size as the –NH– in the amide moiety.^25–27^ Isosteric replacement of an amide by an ester in peptides or proteins has been shown to alter the conformational properties and biological activities.^27^ The amphiphilic polymers were synthesized using a post-functionalization approach (Scheme S1†). Most of the antibacterial polymers reported in literature make use of synthesizing the polymers from their respective cationic and hydrophobic monomers. However, we use the approach of post-functionalization of the polymer to generate amphiphilic polymers. The cationic charge density given by the degree of quaternization was calculated by ^1^H NMR analysis as described previously^28–31^ and found to be in the range of 90–95% for all the polymers. This shows that the cationic charge density was more or less constant in all the polymers which facilitated the comparison between them. The polymers shown in Fig. 2 are represented as AC3P and EC3P for poly(isobutylene-*alt-N*-(*N*′,*N*′-dimethylaminopropyl)-maleimide) (PIBMI) based quaternized amide (A) and ester (E) appended alkyl side chains respectively. A control polymer, HexP (Fig. 2) without hydrogen bonding moieties in the side chains has also been prepared.

### Antibacterial activity and mammalian cell toxicity

**Fig. 3:**
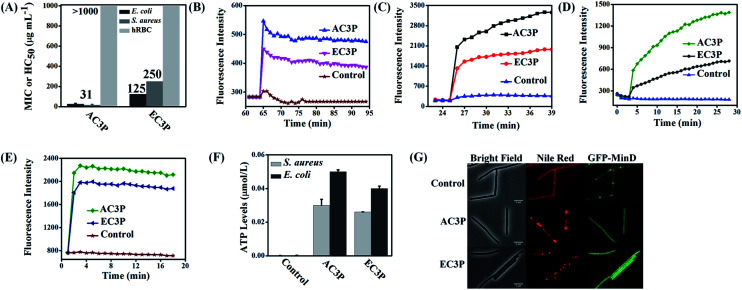
Effect of isosteric substitution on biological activity of cationic-amphiphilic polymers. (A) Antibacterial activity and hemolysis of amide and ester polymers; membrane depolarization of polymers measured through increase of fluorescence of membrane potential sensitive dye, DiSC_3_(5) against *E. coli* (B) and *S. aureus* (C); membrane permeabilization of polymers measured through increase of propidium iodide (PI) fluorescence against *E. coli* (D) and *S. aureus* (E); leakage of ATP levels after treatment for 15 min against *E. coli* and *S. aureus* (F). Relative ATP levels (μmol L^−1^) were determined by subtracting the background ATP levels from the test sample ATP levels; (G) effect on bacterial cell division after the treatment of amide and ester polymers. Mis-localization of cell division-regulating protein, MinD in *B. subtilis* 168 strain carrying a GFP-MinD fusion. Distorted Nile red fluorescence shows alteration in membrane lipid staining after treatment with the polymers (scale bar = 5 μm).

To understand the effect of isosteric replacement, we investigated the antibacterial activity and hemolysis of the polymers. Amide polymer (AC3P) has potent antibacterial efficacy with MIC of 31 μg mL^−1^, whereas its corresponding ester polymer (EC3P) was weakly antibacterial with MIC = 125–250 μg mL^−1^ against both *E. coli* and *S. aureus* (Fig. 3A).

Antibacterial activity of cationic polymers has been shown to be affected by the type of culture media used.^32^ To confirm the fact that the differences in antibacterial activity of these polymers were indeed due to the structural variations, experiments were carried out in chemically defined media. Minimum bactericidal concentration (MBC) in chemically defined media was found to be 2 μg mL^−1^ and 3 μg mL^−1^ for AC3P whereas EC3P showed 16 μg mL^−1^ and >200 μg mL^−1^ against *S. aureus* and *E. coli*, respectively (Table S1†). This observation reiterates the fact that the effect of isosteric replacement on antibacterial activity is indeed due the differences in their chemical structure and not the culture media conditions. Amide and ester polymers had HC_50_ > 1000 μg mL^−1^ (Fig. 3A). The control polymer, HexP though displayed potent antibacterial efficacy but was highly toxic to hRBCs (HC_50_ = 30 μg mL^−1^) leading to low selectivity (HC_50_/MIC) (Table S1†). However, higher selectivity was obtained of >16–32 was obtained for AC3P whereas the ester counter parts, EC3P had low selectivity of >2–8 against both *E. coli* and *S. aureus* (Table S1†).

These results suggested that the HexP without any H-bonding moieties in the side chains is highly toxic whereas AC3P is less toxic to mammalian cells indicating the importance of amide moiety. On the other hand, EC3P was neither antibacterial nor toxic to mammalian cells. Therefore, it appears that the amide side chain containing polymers selectively kill bacteria compared to their ester counterparts sparing the mammalian cells.

### Polymers dissipate membrane potential and show membrane permeabilization

The membrane-active properties of antibacterial polymers were studied in detail to better understand the effects of isosteric replacement towards antibacterial activity and mammalian system toxicity. Membrane disruption has been shown to be one of the main mechanisms of action of natural AMPs.^7^ We assessed whether the bacterial cell membranes were depolarized by the polymers using the membrane potential sensitive dye, DiSC_3_(5) (3,3′-dipropylthiadicarbocyanine iodide) against *E. coli* and *S. aureus* at 25 μg mL^−1^. AC3P has higher depolarization than the corresponding ester, EC3P (Fig. 3B and C) which could be correlated with the low antibacterial activity of the ester against *E. coli* and *S. aureus*. Kinetics of membrane permeabilization was studied by measuring the uptake of the fluorescent probe propidium iodide (PI) against *E. coli* and *S. aureus* at 25 μg mL^−1^. Amide containing polymer has higher ability to permeabilize the bacterial cell membrane than ester polymer (Fig. 3D and E). HexP had lower ability than AC3P and higher ability than EC3P with respect to the membrane-disruptive activity in bacteria (Fig. S1†).

### Polymers cause leakage of ATP levels

Dissipation of membrane potential leads to limiting the energy required for metabolic processes in bacteria.^15^ Extracellular ATP levels in *E. coli* and *S. aureus* were measured after treatment with AC3P and EC3P using the luciferin–luciferase bio-luminescence assay to understand the energy limitation conditions. AC3P (50 μg mL^−1^) showed higher release of ATP levels compared to its ester counterpart, EC3P (50 μg mL^−1^) in both *E. coli* and *S. aureus* (Fig. 3F). This supports the fact that the interaction of the amide and ester polymers with the bacterial membrane is different resulting in variable release of ATP levels.

### Mis-localization of cell division proteins

It has been shown that membrane potential is important for bacterial cell division.^33^ Since the amide and ester polymers dissipate the membrane potential, we examined the localization of three essential cell division proteins, MinD, MreB and FtsZ using a *B. subtilis* 168 strain carrying the relevant GFP reporter fusions. As shown in Fig. 3G and S2,† within 10 min of addition of AC3P and EC3P (both at 25 μg mL^−1^), the dissipation of membrane potential resulted in the complete delocalization of MinD, MreB and FtsZ. CCCP (carbonyl cyanide *m*-chlorophenyl hydrazone, 100 μM) had been used as a positive control to see the effect of dissipating the membrane potential on delocalization of MinD (Fig. S2A†). The dissipation of membrane potential also affected the membrane staining by the fluorescent probe, Nile red. The bright fluorescent membrane foci observed upon addition of the polymers probably represents membrane blobs which are a consequence of rapid loss of cell turgor. These results suggested that due to the dissipation of membrane potential in the presence of these cationic polymers key morphological proteins get mis-localized.

### Polymers affect membrane fluidity/viscosity

**Fig. 4:**
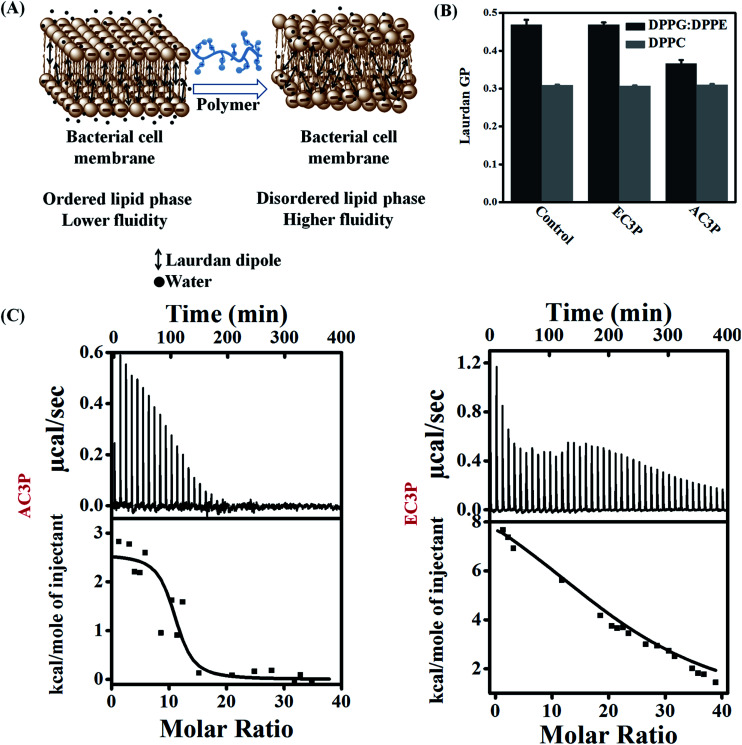
Mechanistic studies to understand the interactions of amide and ester polymers with bacterial and mammalian model lipid bilayers. (A) Membrane hydration was determined using perturbations in Laurdan dye fluorescence due to the result of hydration of the lipid bilayer and quantified by calculating generalized polarization (GP); (B) membrane hydration of lipid bilayers at 37 °C consisting of DPPG : DPPE (88 : 12) and DPPC treated with amide and ester polymers mimicking the bacterial and mammalian membranes, respectively; (C) isothermal titration calorimetry (ITC) thermograms of polymers with DPPG : DPPE (88 : 12). The lipid suspensions (1 mM) were injected into 50 μg mL^−1^ of polymers at 37 °C in 10 mM HEPES and 0.14 M NaCl buffer.

To emphasize the fact that the interaction of the polymers is indeed with the cell membrane, we used model lipid bilayers. Liposomes were made using DPPG : DPPE (88 : 12) and DPPC mimicking the bacterial and human erythrocyte membranes respectively with Laurdan (6-dodecanoyl-2-dimethylaminonaphthalene), a hydrophobic dye encapsulated in them (Fig. 4A).^34^ Due to its structure, dipole moment and its fluorescence characteristics, Laurdan is very useful in studies about lipid bilayer dynamics. Laurdan detects changes in the membrane-phase properties through its sensitivity to the polarity of environment in the lipid bilayer. Polarity changes cause shifts in Laurdan emission spectrum. Interaction of these polymers with the membrane can result in membrane hydration that can be monitored using perturbations in Laurdan dye fluorescence and quantified by calculating general polarization (GP = (*I*_440_ − *I*_490_)/(*I*_440_ + *I*_490_)).^34^ Lower GP indicates higher fluidity (lower viscosity) of the membrane. The Laurdan GP was calculated after the treatment of DPPG : DPPE (88 : 12) and DPPC lipid bilayers with the amide (AC3P) and ester (EC3P) polymers (lipid : polymer = 7.4 : 1) (Fig. 4B). GP for AC3P for DPPG : DPPE lipid system is low compared to the untreated bilayer. On the other hand, ester polymer, EC3P did not show any reduction in GP for DPPG : DPPE compared to the untreated lipid bilayer. These results suggest that an amide polymer is more effective than an ester polymer suggesting the importance of amide moiety. For DPPC bilayer, both polymers showed no reduction in GP compared to the untreated bilayer (Fig. 4B). We believe that the observed changes in GP might be due to a multitude of factors such as membrane hydration, penetration of side chains into the lipid bilayers and the influence of hydrogen bonding in the local polarity of the probe. These observations suggested that AC3P displayed selective interaction with the bacterial cell membrane whereas its ester counterpart, EC3P was devoid of selectivity between bacterial and mammalian cell membranes.

### Thermodynamic of membrane interactions

To delineate the molecular understanding of interactions between the model lipid bilayers and polymers, we used isothermal titration calorimetry (ITC) for obtaining the thermodynamics of interactions. DPPG : DPPE (88 : 12) or DPPC model lipid bilayers were injected into the polymer solution, *i.e.* by gradually reducing the concentration of the free polymer in the solution (lipid : polymer = 30 : 1). The interaction of the polymers with a DPPG : DPPE (88 : 12) model lipid bilayer was found to be an entropy-driven endothermic process. AC3P had a complete and spontaneous entropy-driven interaction with the bacterial model lipid bilayer (within 20 injections) having Δ*G* = −11.03 kcal mol^−1^ but with a lower positive Δ*S* = 43.9 cal mol^−1^ K^−1^ (Δ*H* = 2.57 kcal mol^−1^) (Fig. 4C). The control polymer, HexP had Δ*G* = −9.8 kcal mol^−1^, Δ*H* = 10.75 kcal mol^−1^ and Δ*S* = 66.3 cal mol^−1^ K^−1^ but did not possess complete interaction (Fig. S3†). On the other hand, EC3P did produce an endothermic heat of reaction but had an incomplete interaction with DPPG : DPPE (88 : 12) model lipid bilayer even after 40 injections (Fig. 4C). These results suggested that AC3P had faster, more spontaneous and complete interaction with the bacterial model lipid bilayer compared to the ester polymer stressing the advantage of amide functionality. With the zwitterionic mammalian model lipid bilayer, DPPC, none of the cationic polymers (lipid : polymer = 30 : 1) displayed any detectable enthalpy changes under the experimental conditions and only the small, nearly constant, and exothermic heat flows suggest a weak or no interaction between the polymers and the lipid bilayer (Fig. S3†). Positive entropy changes (Δ*S*) suggest the “hydrophobic effect” explained by the loss of water as the amphipathic molecule enters the lipid bilayer.^35–37^ More importantly, membrane-active pore formation/perturbation is also an endothermic entropy-driven process. We believe that culmination of endothermic entropy-driven processes such as the hydrophobic effect and membrane perturbation explain the dominating interactions of these polymers with the lipid bilayers. AMPs such as mellitin^35^ and PGLa^36^ were also found to interact with bacterial cell membranes by endothermic entropy-driven processes such as the hydrophobic effect and membrane pore formation. These results also correlate with higher membrane-active properties and potent antibacterial efficacy of amide containing polymers than their ester counterparts.

### Molecular dynamics (MD) simulations

**Fig. 5:**
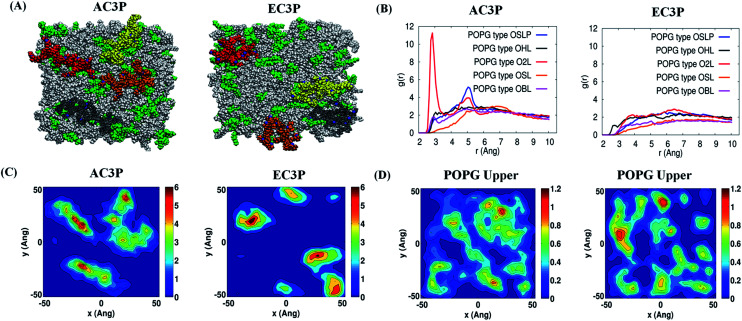
Atomistic molecular dynamics (MD) simulations of the polymers and POPE : POPG (7 : 3) model lipid bilayer after 150 ns simulations. (A) *X*–*Z* (top) view of the polymers and lipid bilayer. The POPE and POPG lipid molecules are colored in white and green respectively. The polymer chains (four) of amide and ester polymers are shown in red, yellow, orange and grey. (B) *g*(*r*) plots reflecting hydrogen bonding interactions of –NH– of the amide moiety and their absence in –C=O of the ester moiety in the amide and ester polymers respectively with the POPG region of the lipid bilayer. (C) 2-D number density plots of polymers and (D) POPG molecules in the upper leaflet of the lipid bilayer.

Experimental investigations such as ITC and Laurdan studies did provide the variable interactions of amide and ester polymers with the bacterial membranes. However, to gain insight into the nature of interactions that play role in the binding of amide and ester polymers with the bacterial membranes we resorted to all atom molecular dynamics (MD) simulations. The interactions were modelled using a mixture of (POPE : POPG = 7 : 3).^38^ Each polymer has four representative polymeric chains with 12 monomers each (monomer is an alternating *N*-alkylmaleimide and isobutylene moieties) (Fig. 5) which were simulated for their interaction with the lipid bilayer. Results after 150 ns simulations of amide and ester polymers with the lipid bilayers showed interesting differences in their membrane-bound conformations as well as their interactions with the lipid head group atoms. The amide polymer (AC3P) showed greater propensities to adopt extended conformations at the lipid interface, thus maximizing the number of contacts with lipid head groups, while the ester (EC3P) polymer adopted considerably more compact conformations (Fig. 5A, S4 and S5†). Conformational changes were found to be induced to a considerably great extent in amide polymer than ester counterpart upon interaction with the lipid bilayers (Fig. S4 and S5†). Even HexP did not have appreciable conformational changes compared to the amide polymers upon interaction with the lipid bilayer (Fig. S5†). These results suggest the induced conformational changes in amide polymers similar to AMPs for *e.g.* induced secondary structure formation upon interaction with the bacterial membranes.^4^

**Fig. 6:**
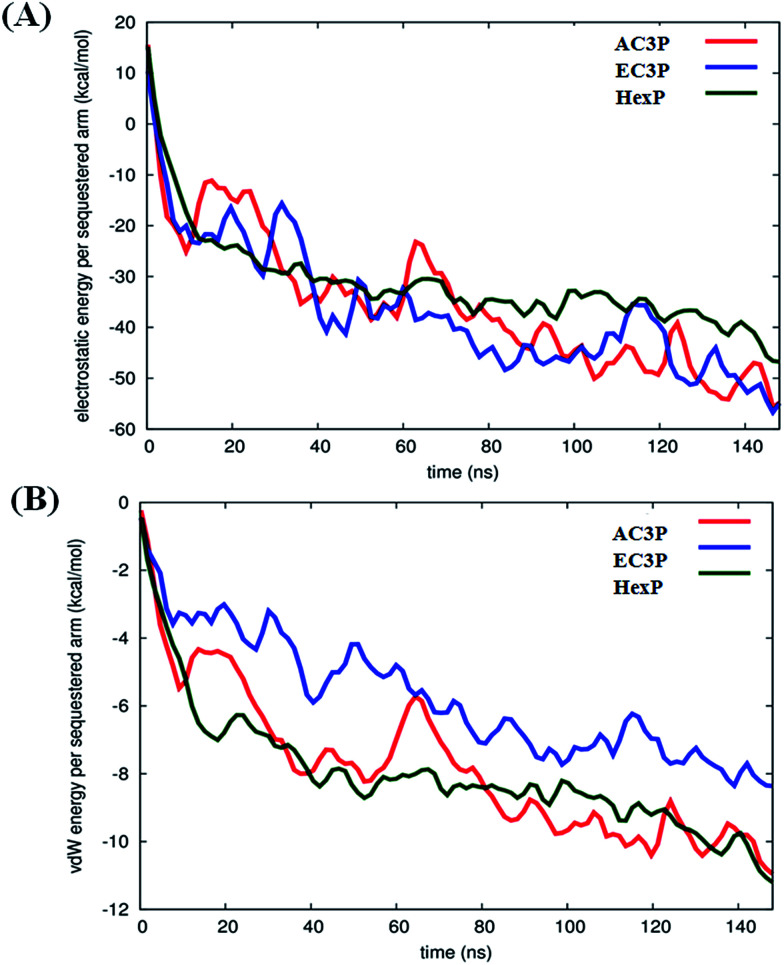
Interaction energies of polymers with the lipid bilayer (POPG : POPE) after 150 ns of simulations. The electrostatic (A) and van der Waals' (vdW) (B) interaction energies have been computed in per sequestered side chain basis to keep them in same footing since various polymers have different number of sequestered side arms after 150 ns of simulation.

Such differences in conformations indicate that these isosteric amide and ester polymers differ in the favourability of their interactions with bilayer lipids. To investigate the same, their interaction energies with the lipid bilayers were computed. The interaction energies have been computed in per sequestered side chain basis to keep them in same footing, since various polymers have different number of sequestered side arms after 150 ns of simulations. It can be seen from Fig. 6A that the electrostatic interaction energies (more negative value indicating stronger interactions) of all the polymers were statistically equal. This is expected since the cationic charge density is constant in the present approach. However, the van der Waals' (vdW) interaction energies, were more negative (hence more attractive) for AC3P and HexP but were statistically less for EC3P (Fig. 6B). van der Waals' (vdW) interaction energies are likely to incorporate a greater signature of interactions of partitioned side chains of these polymers with the hydrophobic lipid chains. This is consistent with the extent to which the polymers have been inserted into the lipid bilayer (Fig. S4 and S5†).

The differences in the ability of isosteric polymer species to form hydrogen bonds with the lipid head group atoms were probed through direct computations of number of hydrogen bonds. The positional order in the spatial distribution of hydrogen bond forming lipid head group atoms in the neighbourhood of amide and ester moieties has also been calculated. Conventionally, in atomistic MD simulations, hydrogen bonds are calculated using geometric criteria and the same was used in the present cases (donor–acceptor distance ≤ 4.0 angstrom (Å) and donor–H–acceptor angle ≤ 60°). The amide polymers have displayed a greater propensity to form hydrogen bonds compared to their ester counterparts, both in the overall number of hydrogen bonds formed and the number of sequestered side arms observed to be involved in the formation of said bonds (Table S2†). The propensity of atomic species' to act as hydrogen bonded partners is portrayed in their radial distribution function or two point density correlation function (*g*(*r*)). Radial distribution functions have been computed between the amide and ester groups' hydrogen bond formers and the same from lipid head group atoms and are interpret in the following as their relative affinities in forming hydrogen bonds. The amide group (–NH– and –C=O moieties) of the amide polymers interacted through strong hydrogen bonds with the oxygen atoms of the phosphate head groups of the POPG lipid molecules as seen in Fig. 5B and S6.† The amide groups also form hydrogen bonds, albeit weaker, with the oxygen atoms of phosphate head groups and ammonium moieties of POPE lipid molecules (Fig. S6†). On the other hand, the carbonyl group of their ester counterparts has no strong interactions with POPG molecules (Fig. 3B and S7†), and form weak hydrogen bonds with ammonium moieties of only POPE. HexP forms the lowest number of hydrogen bonds probably due to the backbone imide groups (Table S2†). These results suggest that the amide polymers have strong interactions whereas the ester polymers are devoid of any preferentially favourable interactions with the negatively charged POPG lipids. These results also support the observation of more stretched conformations of amide polymers.

The discernible differences in lipid–amide polymer and lipid–ester polymer interactions described above are further reflected in the relative ability of isosteric polymers in inducing structural re-organization of the lipid bilayer. The number density distribution of polymers and POPG molecules in the upper leaflet is shown in Fig. 5C and D and S8.† It can be seen very clearly that the location of amide polymers and POPG clusters are well correlated and the amide polymers seem to have the ability to reorganize the upper leaflet of the bacterial membrane through favourable interactions between the partitioned side chains and the POPG lipid molecules. These MD simulations support the potent antibacterial efficacy of amide polymers due to the culmination of various stronger interactions with the bacterial cell membrane than their ester counterparts. Random methacrylate polymers bearing primary ammonium groups by Kuroda and co-workers with cationic charge and hydrophobicity have been speculated to show the capability of hydrogen bonding with the phosphate lipid head groups.^20^

### Raman spectroscopy

To gain further insight into the hydrogen bonding interactions of polymers with lipid molecules, Raman spectroscopic studies were performed. The organic solution of the lipid + polymer was drop-casted onto a substrate, dried and studied using a Raman spectrophotometer. The full Raman spectra are provided in ESI (Fig. S9†). Since the interest is in the specific interaction of polymers (AC3P, EC3P, HexP) with the bacterial lipid (DPPG), we concentrate on the modes related to the phosphate region of the lipid (1040–1150 cm^−1^) and the amide (–CONH–) and the ester (–COO–) regions in the polymers (1600–1800 cm^−1^).

**Fig. 7:**
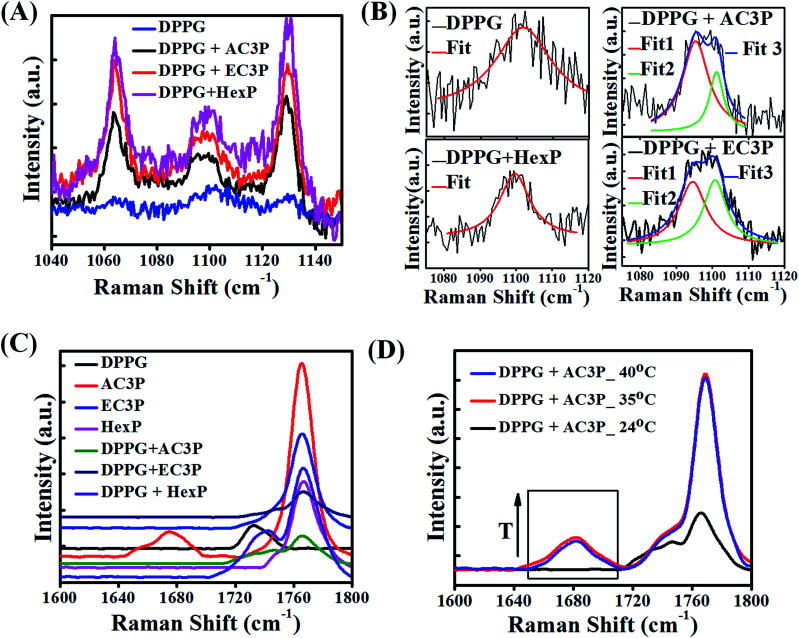
Polymer interactions with bacterial membrane lipid, DPPG using Raman spectroscopy. (A) Lipid phosphate vibrational modes 1064 cm^−1^, –P–O– (DPPG head group), 1100 cm^−1^, PO_2_^−^ and 1129 cm^−1^, –P–O– (DPPG tail group) DPPG and polymer + DPPG indicate softening (decrease in frequency) of all three modes. (B) Splitting of PO_2_^−^ mode into a doublet (loss of degeneracy) due to strong interactions of amide polymer with DPPG, followed by weak interactions of ester polymer with DPPG and weaker interaction of HexP with DPPG. (C) Spectra in the region of 1600–1800 cm^−1^ showing side chain amide I vibration (1680 cm^−1^) and the side chain ester C=O vibrational mode (1740 cm^−1^), long chain ester C=O of DPPG (black curve) and polymer backbone C=O (imide) at around 1770 cm^−1^. Upon interaction with DPPG, amide I mode (1680 cm^−1^) of AC3P (red curve) disappears in DPPG + AC3P (green curve). (D) Temperature dependent spectra (arrow showing increase in temperature, *T*) of DPPG + AC3P showing the emergence of amide I mode at high temperatures showing the strong hydrogen bonding between AC3P and DPPG.

As shown in Fig. 7A, 1064 cm^−1^ vibrational mode is due to –P–O– (DPPG head group), 1100 cm^−1^ is due to PO_2_^−^ and 1129 cm^−1^ is due to –P–O– (DPPG tail group) (blue curve) for DPPG.^39^ Upon polymer interaction, all the three modes showed softening (decrease in frequency) indicating that all the three polymers interact with phosphate region of DPPG. It is interesting to note that 1100 cm^−1^ vibrational mode splits into doublet upon interacting with AC3P and EC3P (Fig. 7B) along with softening. O=P–O^−^ has the ability to resonate, hence the Fermi resonance (expected in AB_2_) doublet does not exist in PO_2_^−^ of DPPG (Fig. 7B). But upon addition of all three polymers, the electrostatic interaction with DPPG would affect the otherwise degenerate PO_2_^−^ vibration leading to splitting. HexP has less chance for hydrogen bonding with DPPG and hence the non-directional electrostatic interaction is weaker with no splitting of PO_2_^−^ vibration (Fig. 7B). The hydrogen bonding ability of AC3P and EC3P with DPPG gets the cationic nitrogen localized in space leading to strong interactions with the PO_2_^−^ group resulting in its splitting. The lower frequency mode is due to the PO^−^ group (1096 cm^−1^) and the higher frequency mode is due to the P=O group (1100 cm^−1^). Interestingly, the relative intensities of the two peaks in doublet is higher in AC3P + DPPG than in EC3P + DPPG suggesting the strong interactions of amide polymer with the lipid compared to the ester polymer (Fig. 7B).

The polymer region (1600–1800 cm^−1^) of the spectra as shown in Fig. 7C is dominated by the side chain amide I vibration (1680 cm^−1^) and the side chain ester C=O vibrational mode (1740 cm^−1^).^39,40^ This region is affected by the presence of a strong signature from the long chain ester C=O of DPPG (black curve Fig. 7C) and also from the polymer backbone C=O (imide) at around 1770 cm^−1^. It is interesting to see that amide I mode (1680 cm^−1^) disappears in DPPG + AC3P (green curve Fig. 7C) and looks very similar to the DPPG + EC3P spectrum (dark blue curve Fig. 7C). This is very important because it shows there is a strong hydrogen bonding between the amide moiety of AC3P and DPPG. It is interesting to see that the spectrum for DPPG + HexP (purple curve Fig. 7C) more or less looks like the convolution of DPPG (black curve, Fig. 7C) and HexP (magenta curve Fig. 7C). The long chain lipid ester (1740 cm^−1^) peak has shifted showing some weak interaction between DPPG and HexP as observed earlier.

In order to confirm the hydrogen bonding, the Raman spectra for DPPG + AC3P was recorded on heating up to 40 °C (phase transition temperature (*T*_m_) of DPPG is 41 °C) (Fig. 7D). The emergence of amide (1680 cm^−1^) band above 35 °C suggested the breaking of hydrogen bond. The strong 1740 cm^−1^ also shows the emergence of long chain ester C=O vibration of the lipid. Similar experiments in the DPPG + EC3P spectrum bring the emergence of a strong 1740 cm^−1^ similar to DPPG + AC3P indicated the breaking of ester polymer C=O hydrogen bond with DPPG (Fig. S10†). Heating effect on the 1100 cm^−1^ mode showed no changes in both DPPG and DPPG + HexP, but in DPPG + AC3P as well as DPPG + EC3P, the splitting still persisted suggesting that the molecules are still weakly hydrogen bonded and in position (Fig. S10†).

The above results clearly suggests that the difference between the interaction of AC3P and EC3P with DPPG is that the amide provides strong hydrogen bonding interactions with DPPG compared to EC3P probably through the two hydroxyl groups of the DPPG head group. These results also correlated with MD simulations which showed the hydrogen bonding interactions of amide polymers with the hydroxyl and phosphate oxygen atoms of POPG in bacterial model lipid bilayers. Taken together, these data suggested that despite similar electrostatic interactions, the lack of strong hydrogen bonding interactions can render weaker binding of ester polymers with lipid bilayers compared to amide polymers.

## Conclusions

In conclusion, isosteric substitution of ester with amide moiety in cationic-amphiphilic polymers influences the bacterial membrane interactions. With the unique concept of amide and ester moiety bearing polymers, for the first time we provided evidence for the role of hydrogen bonding in bacterial membrane interactions. However, the differences in the binding affinity of the amide and ester polymers with the bacterial membranes might not be solely due to the hydrogen bonding. We believe that this understanding will aid in bacterial membrane specific design of membrane-active molecules in future.

## Experimental section

### Materials

All the solvents were of reagent grade and dried prior to use wherever required. Bromoacetyl-bromide, poly(isobutylene-*alt*-maleic anhydride) (*M*_w_ ∼ 6000 Da, Catalog no. 531278), 3-aminopropyldimethylamine and 1-propyl amine were purchased from Sigma-Aldrich and used as received. 1-Propanol was obtained from Spectrochem (India) and used as received. Culture media and the antibiotics were from HIMEDIA (India) and Sigma-Aldrich respectively. NMR spectra were recorded using Bruker AMX-400 (400 MHz for ^1^H) spectrometer. The chemical shifts (*δ*) are reported in parts per million downfield from the peak for the internal standard TMS for ^1^H NMR. Infrared (IR) spectra of the solid compounds were recorded on Bruker IFS66 V/s spectrometer using KBr pellets. IR spectra of the compounds soluble in low-boiling solvents were recorded with the same instrument using NaCl crystal. Mass spectra were recorded on a Micromass Q-ToF micromass spectrometer. Optical density and absorbance were measured by Tecan InfinitePro series M200 Microplate Reader.

### Bacterial strains, media and growth conditions

Bacterial strains *S. aureus* (MTCC 737) and *E. coli* (MTCC 443 or ATCC 25922) were purchased from MTCC (Chandigarh, India). The antibacterial activity of the polymers was done against both Gram-negative (*E. coli*) and Gram-positive (*S. aureus*) bacteria. *E. coli* was cultured in Luria Bertani broth (10 g of tryptone, 5 g of yeast extract, and 10 g of NaCl in 1000 mL of sterile distilled water while *S. aureus* was grown in nutrient broth (1 g of beef extract, 2 g of yeast extract, 5 g of peptone and 5 g of NaCl in 1000 mL of sterile distilled water). For solid media, 5% agar was used along with above mentioned composition. The bacterial samples were freeze dried and stored at −80 °C. 5 μL of these stocks were added to 3 mL of the nutrient broth and the culture was grown for 6 h at 37 °C prior to the experiments.

Synthesis and characterization are provided in ESI.†

Antibacterial activity, hemolytic activity, cytoplasmic membrane depolarization and permeabilization were performed as described earlier.^28–31^

### Release of ATP levels

Mid-log phase bacteria (∼10^8–9^ CFU mL^−1^) were harvested and washed twice with 10 mM TRIS buffer (pH = 7.5) and were resuspended in the same buffer. Then, 150 μL of bacterial suspension and 50 μL of test drugs were added to the micro centrifuge tube and incubated at 37 °C for 15 min. After 15 min, the bacterial suspension was centrifuged and 50 μL of the supernatant was transferred into a Corning 96 well black plate with clear bottom to find out the released ATP levels using ATP Bioluminescence Assay Kit (Sigma Aldrich) as per the manufacturer's instructions. A standard curve for ATP levels was generated using the ATP standards provided in the kit in the range of 1 × 10^−6^ to 1 × 10^−11^ moles of ATP levels. Relative ATP levels both in the standard curve and the test sample measurement were measured by subtracting the background ATP levels from the test sample ATP levels as per the manufacturer's instructions. All measurements were performed in duplicates using Tecan InfinitePro series M200 Microplate Reader.

### Alteration of cell division proteins^33^

*B. subtilis* strains were cultured in LB medium. Overnight cultures from a single colony were diluted and grown at 30 °C. Xylose (0.1–0.5%) was added to induce the GFP fusion proteins. At OD_600_ ∼ 0.2, 200 μL of cells were incubated with 25 μg mL^−1^ of polymers or positive control (CCCP, 100 μM) for 10 minutes. The last 3 minutes, 1 μg mL^−1^ Nile red (Sigma) was added for membrane staining. Microscope slides were covered with 1% agarose in deionized water. 0.4 μL of cells were spotted onto the agarose layer and excess liquid was allowed to evaporate before a 0.31 mm cover slip (VWR) was placed on top. Cells were observed with a Nikon Eclipse Ti inverted microscope with Nikon plan apo 100× 1.45 oil objective, and Hamamatsu ORCA Flash UBB 3.0 camera. Images were processed using Nikon and ImageJ software.

### Liposome preparation

Lipids (0.5 mM of DPPC or 0.5 mM DPPG : DPPE (88 : 12)) and Laurdan dye (5 μM) were taken in round-bottom glass vials in chloroform. Thin films were made under dry argon gas, and films were dried under vacuum for complete drying. Lipid films were hydrated with 1× PBS (pH = 7.4) for overnight. Hydrated films were then processed for 10 freeze thaw cycles from 70 to 4 °C with intermittent vortexing. Multilamellar vesicles were then sonicated at 70 °C for 15 min to get unilamellar vesicles.

### Membrane hydration^34^

2 mL of Laurdan embedded liposome dispersion (lipid : dye = 100 : 1) containing the polymer solution (lipid : polymer = 7.4 : 1) in PBS (pH = 7.4) was taken in a fluorescence cuvette. The fluorescence emission intensity was measured at 440 nm and 490 nm by using the excitation wavelength at 350 nm. The measurements were performed at 37 °C using Water Peltier system attached PerkinElmer LS-55 Luminescence Spectrometer. Laurdan, a hydrophobic dye detects changes in the membrane-phase properties through its sensitivity to the polarity of environment in the lipid bilayer. Polarity changes are shown by shifts in the Laurdan emission spectrum, which are quantified by calculating the generalized polarization (GP). Then, membrane hydration was determined using perturbations in Laurdan dye fluorescence due to the result of hydration of the lipid bilayer and quantified by calculating general polarization (GP). GP was calculated by using the following equation.GP = (*I*_440_ − *I*_490_)/(*I*_440_ + *I*_490_)wherein *I*_440_ and *I*_490_ represent the fluorescent emission intensity at 440 nm and 490 nm respectively.

### Isothermal titration calorimetry (ITC)

All experiments were performed at 37 °C using isothermal titration calorimeter (Micro Cal Inc.). Reference cell was filled with double distilled water. All the experiments were performed in 10 mM HEPES and 0.14 M NaCl (pH = 7.4) buffer. The liposomal suspensions (DPPC or DPPG : DPPE (88 : 12)) were made in the above buffer at 1 mM and polymers were also dissolved in the same buffer and used at a concentration of 50 μg mL^−1^. The lipid to polymer ratio was kept at 30 : 1. All the samples were degassed before the titrations. The lipid suspensions were taken in the syringe and the polymer solution was filled in the calorimetric cell. The experiment consisted of 40 injections of 5 μL each (2 μL of first injection) with 10 min intervals to ensure that the titration peak returned to the baseline before the next injection was done. Each injection lasted for 10 s and the stirring speed was kept at 307 rpm for homogenous mixing. A background titration was performed under same conditions with the buffer placed in the calorimetric cell instead of the polymer solution. This result was subtracted from each polymeric sample titration to account for the heat of dilution. The change in enthalpy (Δ*H*) and the association constant (*K*_a_) were obtained by fitting the data to one set of sites model using Origin 7 software from Micro Cal Inc. The Gibbs free energy change (Δ*G*) and change in entropy (Δ*S*) were calculated using the following equationΔ*G* = −*RT* ln(55.5*K*_a_) = Δ*H* − *T*Δ*S*wherein, *R* is the universal gas constant (1.986 cal mol^−1^ K^−1^), factor 55.5 is to account for the molar concentration of water and *T* is the absolute temperature in K.

### Atomistic molecular dynamics simulations

Classical atomistic molecular dynamics (MD) simulations were performed using simulation package NAMD (versions 2.8 and 2.9)^41^ to investigate the interaction of the individual polymer species with a model POPE–POPG bacterial membrane patch consisting of 90 POPE and 38 POPG lipid molecules (Table S3†). The bilayer configuration used to construct the polymer-bilayer systems was taken from a fully hydrated, 300 ns equilibrated membrane patch used in our previous published work.^42^ Owing to such long equilibration, the last 50 ns (250–300 ns) of the simulation was used as control study to compute observables in absence of polymers. CHARMM force field CHARMM 36,^43^ optimized to simulate tension less bilayers was used for the lipid (POPE, POPG) molecules and force field parameters for the polymers were derived using the CGenFF program.^44,45^ TIP3P model was used for water and standard CHARMM parameters^46^ for ions (Na^+^, Cl^−^). Equilibrium structures of the 12-monomer length polymers in aqueous environment were obtained by simulating single polymers in 150 mM NaCl solutions for 50 ns. Initial system sizes for such simulations were ∼68 × 68 × 68 Å^3^ containing ∼9500 water molecules. Polymer properties in aqueous environment were computed over the last 30 ns (20–50 ns) of simulation data. The polymer configurations at the end of 50 ns were extracted, replicated and used to construct the polymer-bilayer systems. To construct each of the polymer-bilayer systems, four polymers (of same chemical composition) were placed dispersed in the bilayer plane ∼(12–18) Å away along the bilayer normal from one of the bilayer leaflets (referred to as “upper leaflet”). Additional water molecules were added so as to prevent the polymers from interacting strongly with the other bilayer leaflet (referred to as “lower leaflet”), at least at the initial stages of simulations. Further, Na^+^ and Cl^−^ ions were added to neutralize the excess charges in the systems and set salt concentration at 150 mM. The number of atoms for the polymer-bilayer systems simulated was ∼86 000. These systems were each simulated for 150 ns and stationary properties were computed over the last 20 ns (130–150 ns) of simulation, unless stated otherwise. All simulations were performed under isothermal–isobaric (NPT) conditions at a temperature of 310.15 K. Pressure was maintained at 1 atm using Langevin Piston.^47^ Time step for all simulations was 2.0 fs. Long range electrostatic interactions were computed using particle-mesh Ewald (PME) and Lennard–Jones interactions were truncated beyond 12 Å using a switching function between 10 Å and 12 Å.

### Raman spectroscopy

Measurements were performed using WiTec Raman spectrometer (UHTS600 SMFC) equipped with CCD (CCD-17531). The excitation source was 532 nm with a power of ∼20 mW at the sample. A 60× objective (Nikon make) with a numerical aperture (NA) 0.8 was used for normal Raman and 50× ultra long working distance objective with 0.45 NA was used for temperature dependent studies for focusing the laser and collecting scattered light in 180° back scattering geometry. The typical accumulation times were 600 seconds. Temperature dependent measurements were performed using Linkam cryostage (Linkam Scientific Instrument). 20 μL of methanolic solution (400 μg mL^−1^) of sample was drop coated onto Sapphire substrate, dried under vacuum and studied using a Raman spectrophotometer.

## Supplementary Material

SC-007-C6SC00615A-s001
