# Supplementary material for: Isosteric substitution in cationic-amphiphilic polymers reveals an important role for hydrogen bonding in bacterial membrane interactions
Source: Chem Sci. 2016 Apr 7;7(7):4613–23. doi: 10.1039/c6sc00615a (PMC6016443; doi:10.1039/c6sc00615a)
Supplement: SC-007-C6SC00615A-s001 [file SC-007-C6SC00615A-s001.pdf]

Electronic Supplementary Material (ESI) for Chemical Science.
This journal is © The Royal Society of Chemistry 2016

Supporting Information

Isosteric Substitution in Cationic-amphiphilic Polymers Reveals an Important Role for Hydrogen Bonding in Bacterial Membrane Interactions

Divakara S.S.M. Uppu[a], Mohini M. Konai[a], Upayan Baul[b], Priyank Singh[c], Tjalling Siersma[d], Sandip Samaddar[a], Jiaul Hoque[a], Satyavani Vemparala[b], Leendert W. Hamoen[d], Chandrabhas Narayana[c] and Jayanta Haldar[a,*]

[a]Chemical Biology & Medicinal Chemistry Laboratory, New Chemistry Unit, Jawaharlal Nehru Centre for Advanced Scientific Research (JNCASR), Jakkur, Bangalore 560064, India;
[b]The Institute of Mathematical Sciences (IMSc), C. I. T. Campus, Taramani, Chennai 600113, India;
[c]Light Scattering Laboratory, Chemistry and Physics of Materials Unit, Jawaharlal Nehru Centre for Advanced Scientific Research (JNCASR), Jakkur, Bangalore 560064, India;
[d]Bacterial Cell Biology, Swammerdam Institute for Life Sciences (SILS), University of Amsterdam, Amsterdam 1098 XH, the Netherlands;

[*]Corresponding author. E-mail: jayanta@jncasr.ac.in; Fax: +91-80-2208-2627; Telephone: +91-80-2208-2565.

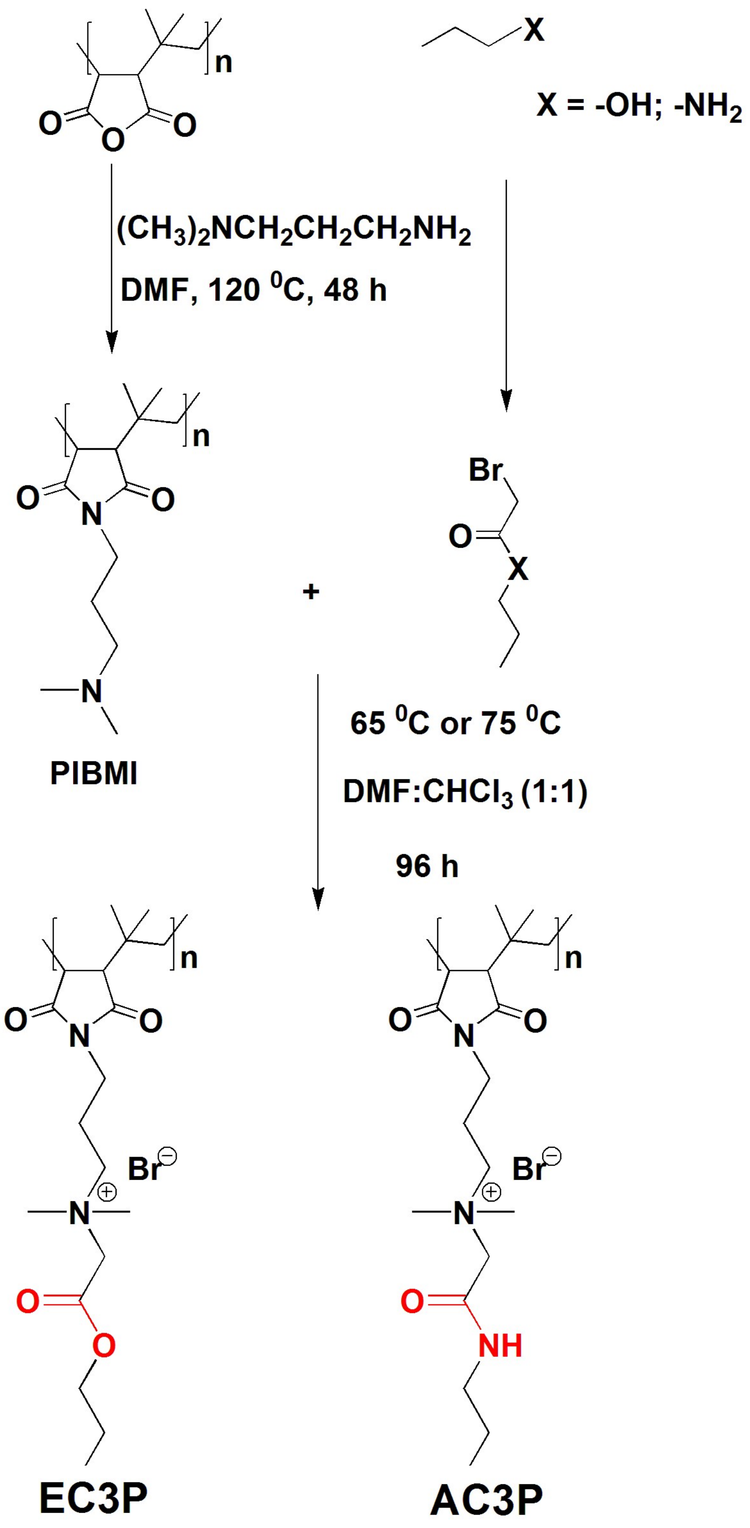

Scheme S1. General synthesis of cationic-amphiphilic polymers.

Table S1. Antibacterial activity, toxicity and selectivity profiles of cationic polymers

Polymer	MIC[a] (μg mL^{-1})		HC_{50}[b] (μg mL^{-1})	Selectivity[c]	
	E. coli	*S. aureus*		*E. coli*	*S. aureus*
AC3P	31	31	>1000	>32	>32
EC3P	125	250	>1000	>8	>4
HexP	7	20	30	4.3	1.5

[a]MIC, minimum inhibitory concentration in cation adjusted Mueller-Hinton broth (CAMHB); [b]HC_{50}, concentration required to cause 50% hemolysis; [c]Selectivity, is defined as HC_{50}/MIC.

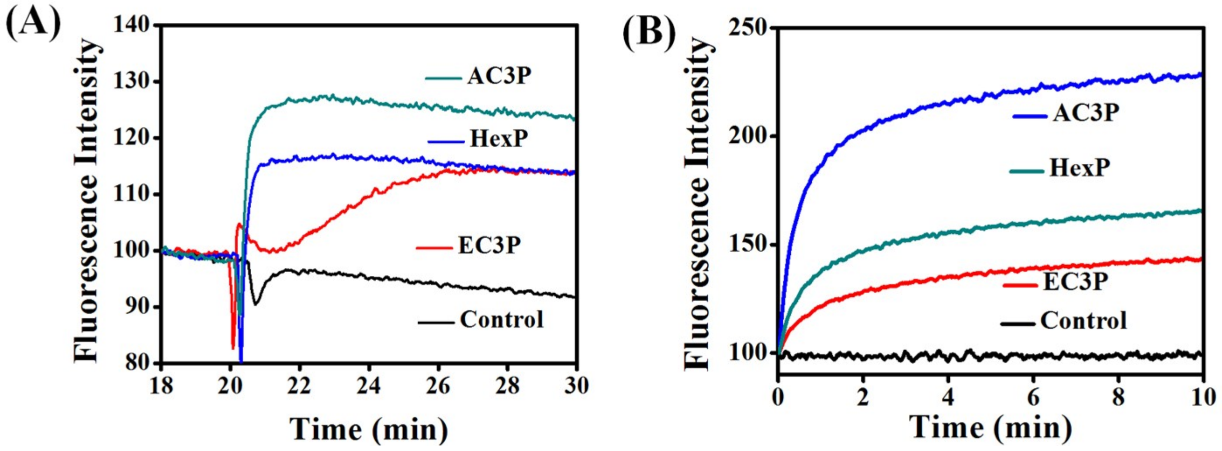

Figure S1. (A) Membrane depolarization and (B) Membrane permeabilization of polymers against *E. coli*.

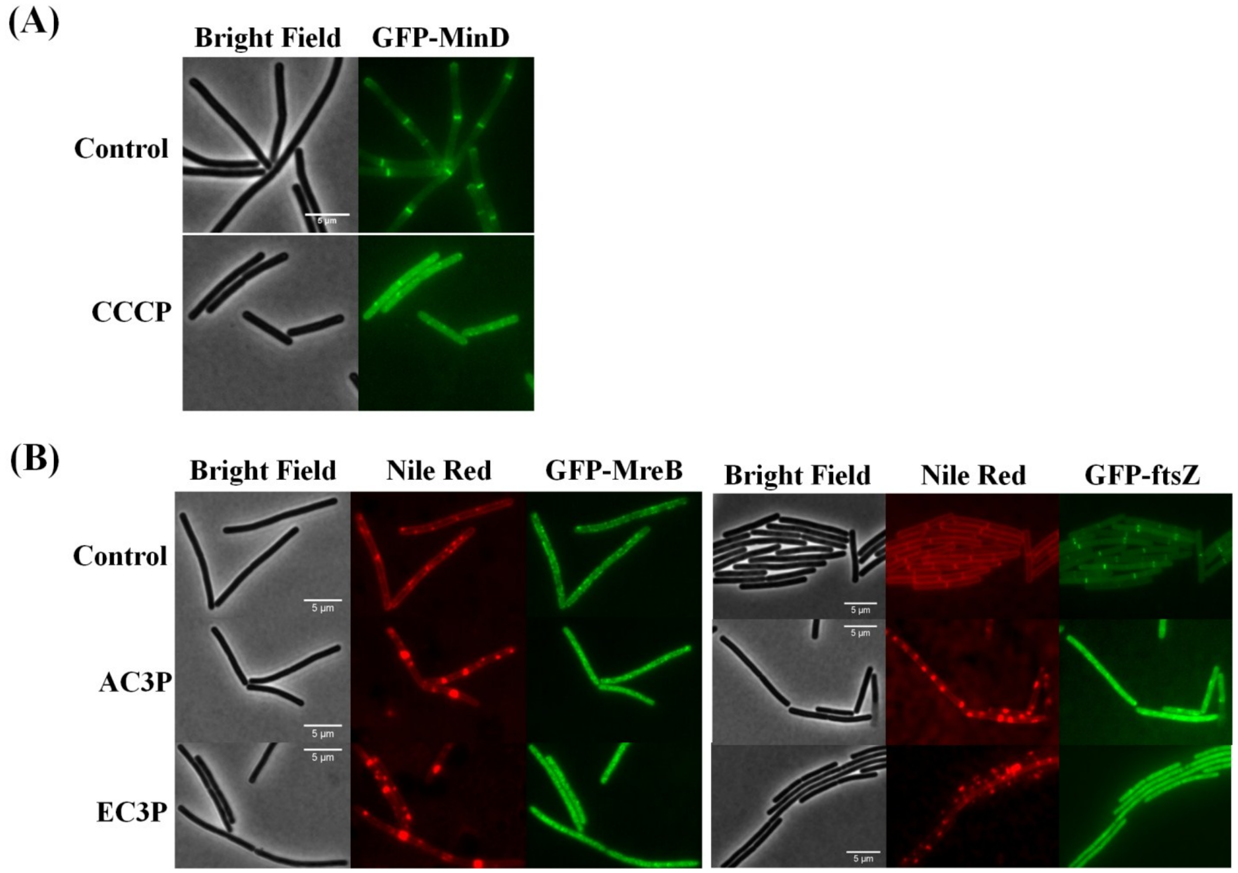

Figure S2. Effect on the bacterial cytoskeleton protein MreB and the cell division protein, FtsZ in *B. subtilis*. Strain carrying a GFP-MreB and FtsZ-GFP fusion were used. Distorted Nile red fluorescence shows alteration in membrane lipid staining after treatment with the polymers. Bacteria were treated for 10 min in the presence of polymers (25 µg mL^{-1}) and positive control (CCCP, 100 µM). Scale bar, 5 µm

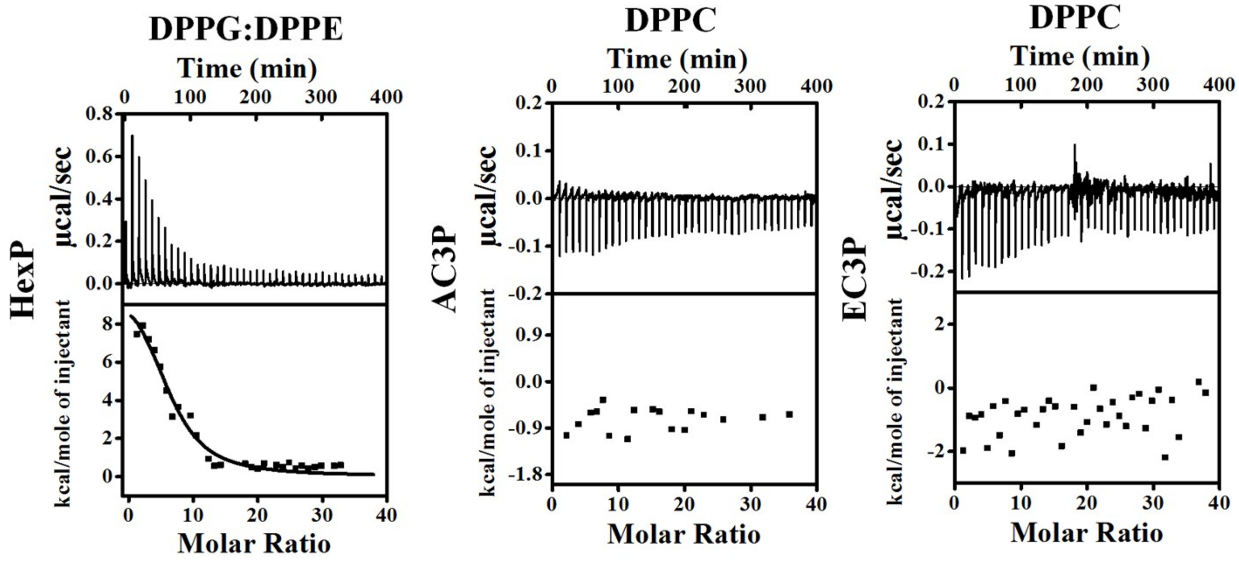

Figure S3. Isothermal titration calorimetry (ITC) thermograms of polymers with DPPG:DPPE(88:12) and DPPC. The lipid suspensions (1 mM) were injected into 50 µg mL^{-1} of polymers at 37 °C in 10 mM HEPES and 0.14 M NaCl buffer.

Table S2. Number of hydrogen bonds formed calculated over the last 20 ns (130-150 ns) of simulations.

Polymer	No. of hydrogen Bonds	No. of side arms involved
AC3P	34280	25537
EC3P	8343	7709
HexP	4035	4000

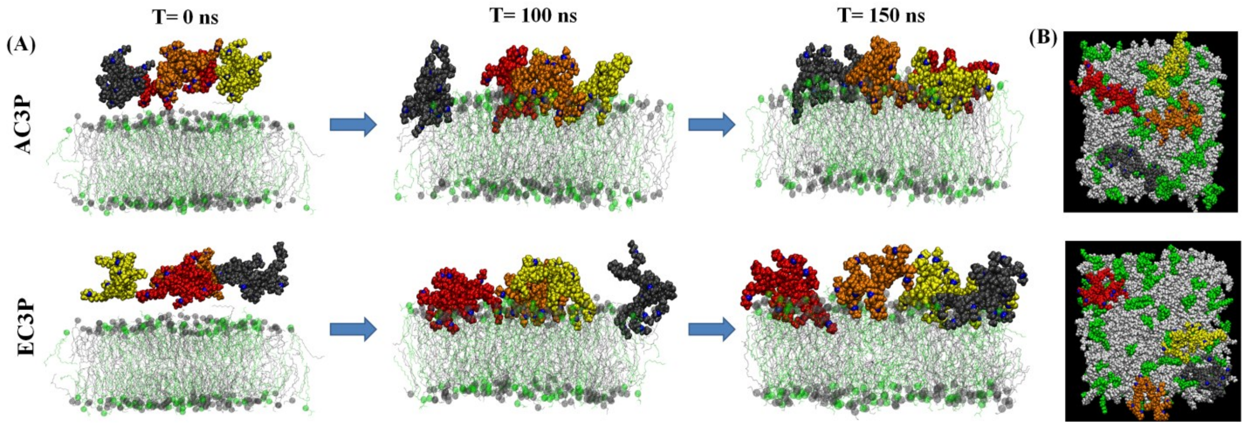

Figure S4. Atomistic molecular dynamics (MD) simulations of the polymers and POPE:POPG (7:3) model lipid bilayer. (A) X-Y (lateral) and (B) X-Z (top) view of the polymers and POPE:POPG lipid bilayer after 150 ns simulations. The POPE and POPG lipid molecules are colored in light grey and green respectively. The polymer chains (four) of amide and ester polymers are shown in red, yellow, orange and dark grey.

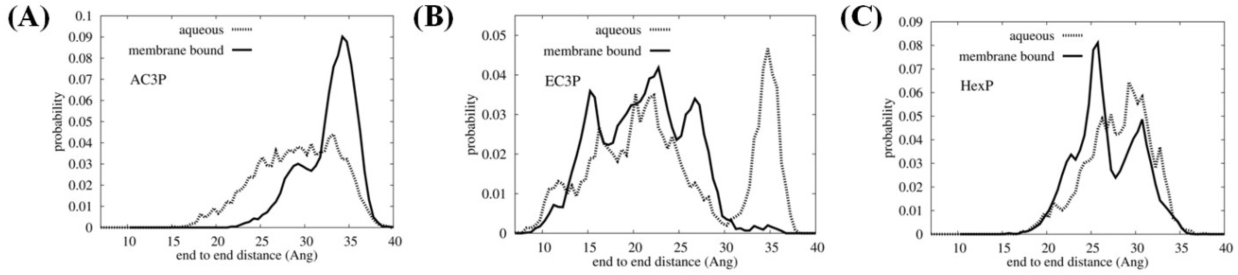

Figure S5. (A) End-to-end distance of all the polymers during the last 30 ns (120-150) ns of simulations with the bacterial lipid bilayers. End-to-end distance of polymer was calculated by comparing the initial conformation (in aqueous phase, without the bilayer) and final conformation (upon interaction with the bilayer).

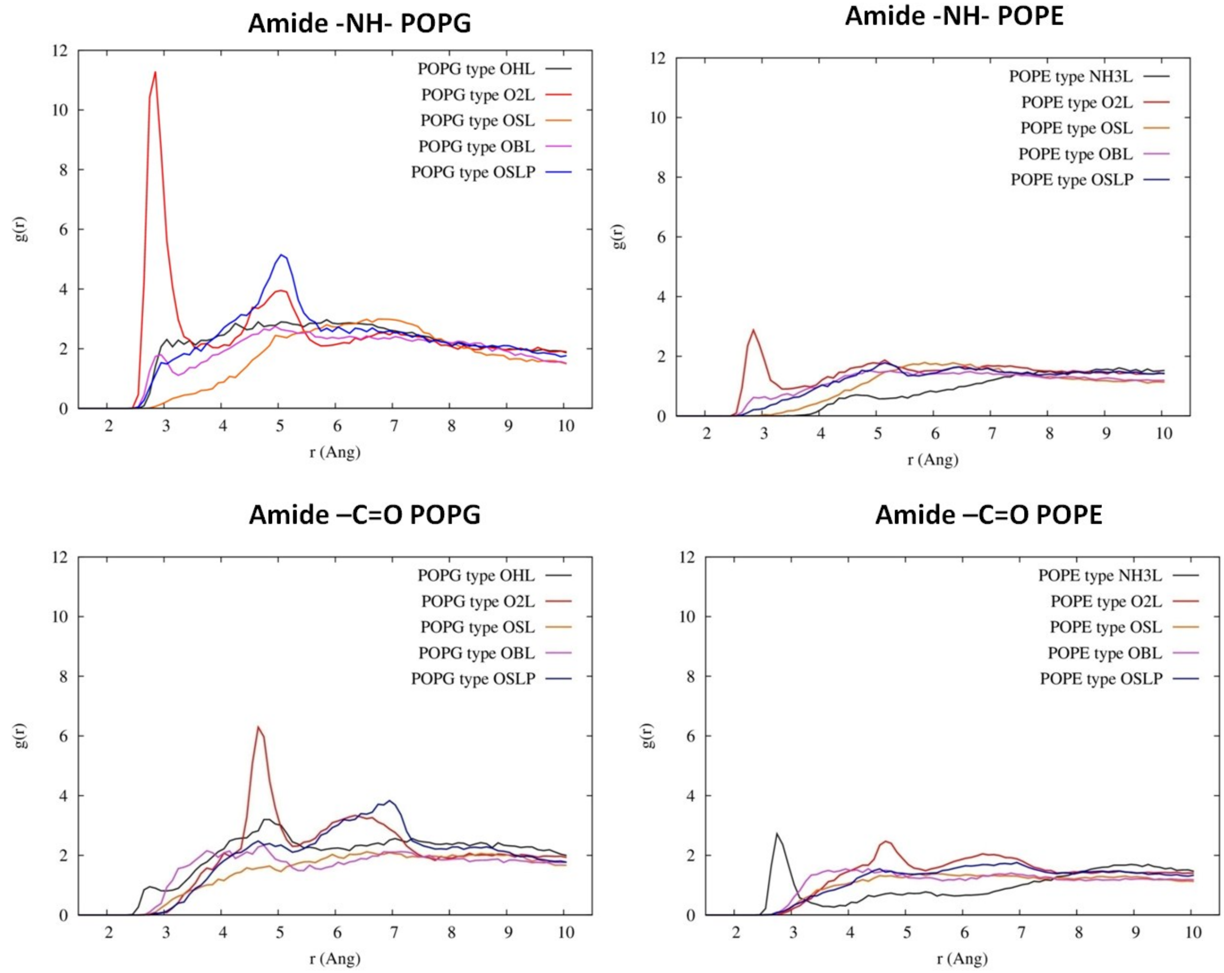

Figure S6. g(r) plots reflecting hydrogen bonding interactions of -NH- and –C=O of the amide moiety of the amide polymer with the lipid bilayer (POPG:POPE) after 150 ns simulations.

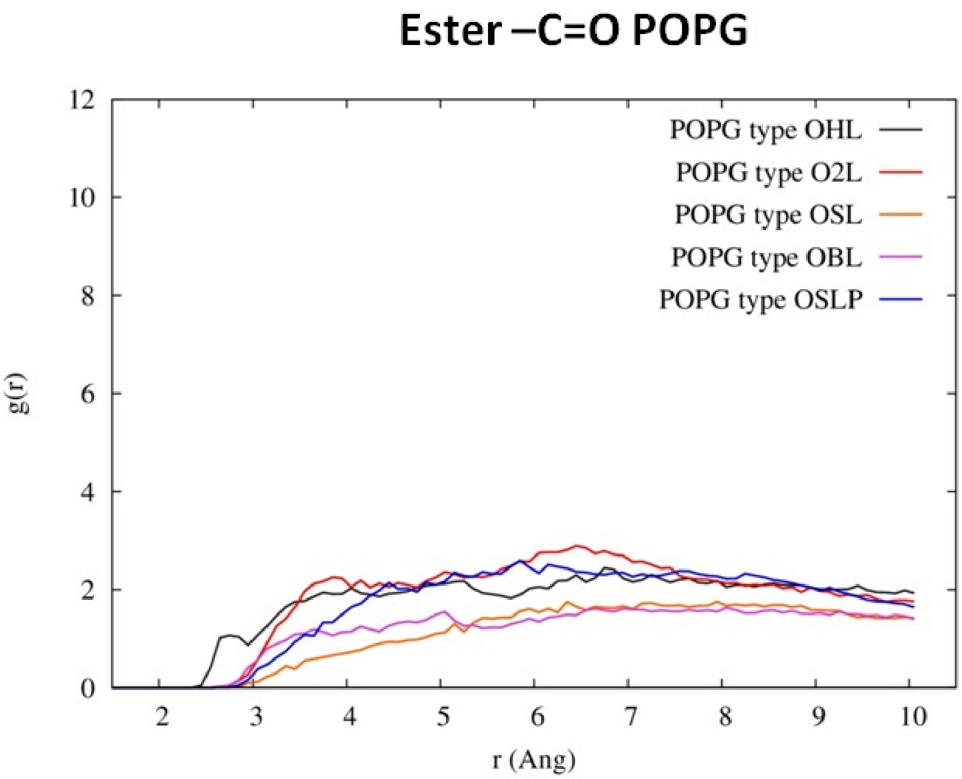

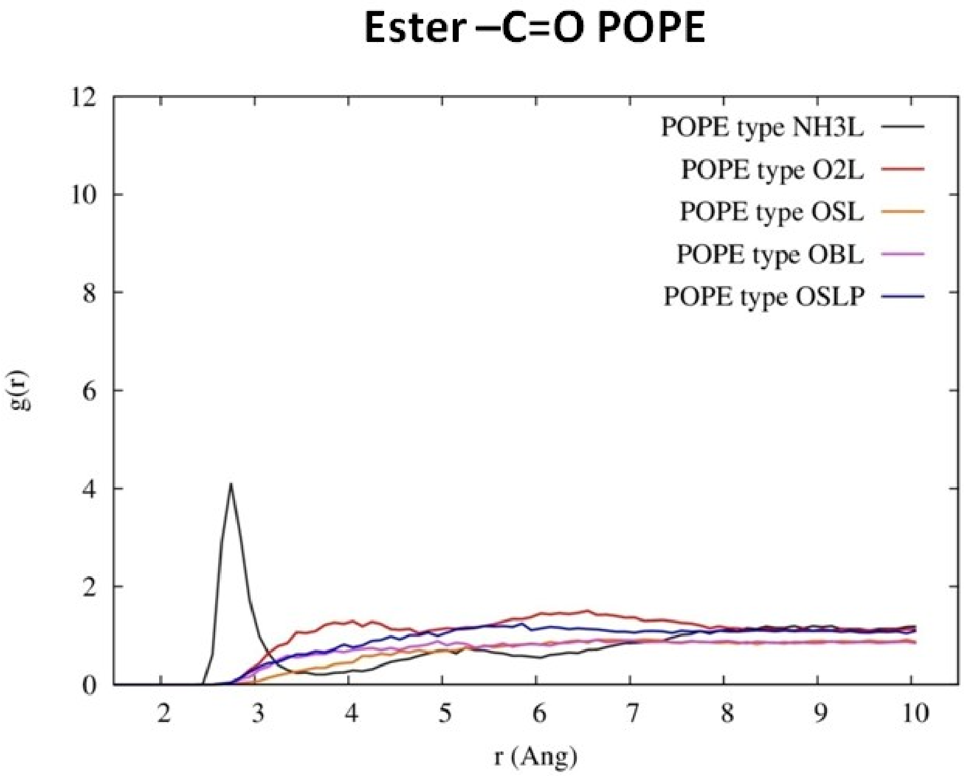

Figure S7. g(r) plots reflecting absence of hydrogen bonding interactions of the –C=O of the ester moiety in the ester polymer with the lipid bilayer (POPG:POPE) after 150 ns simulations.

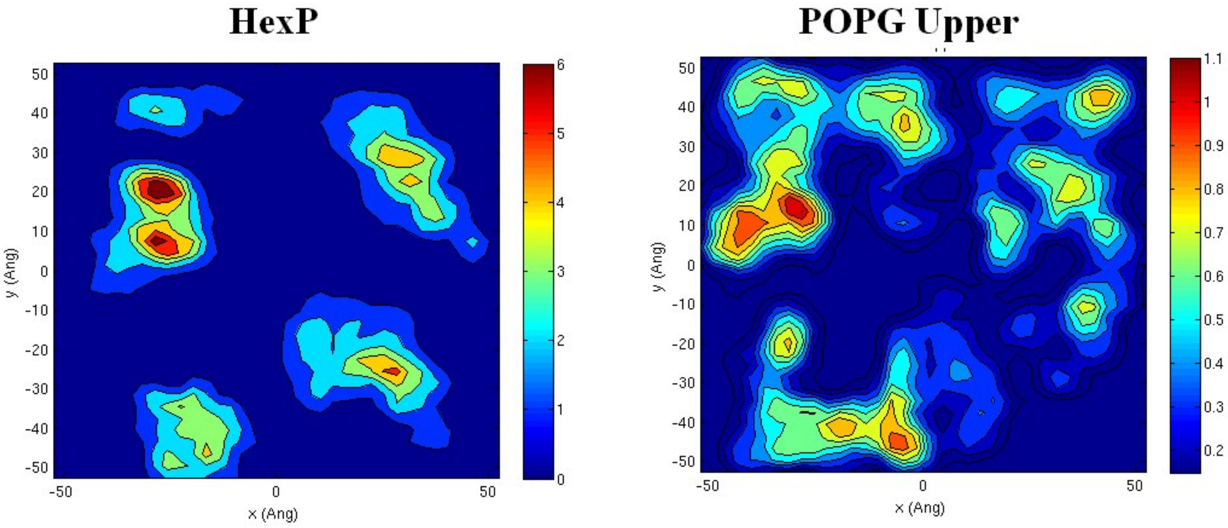

Figure S8. 2-D number density plots of polymer and POPG molecules in the upper leaflet of the lipid bilayer (POPG:POPE).

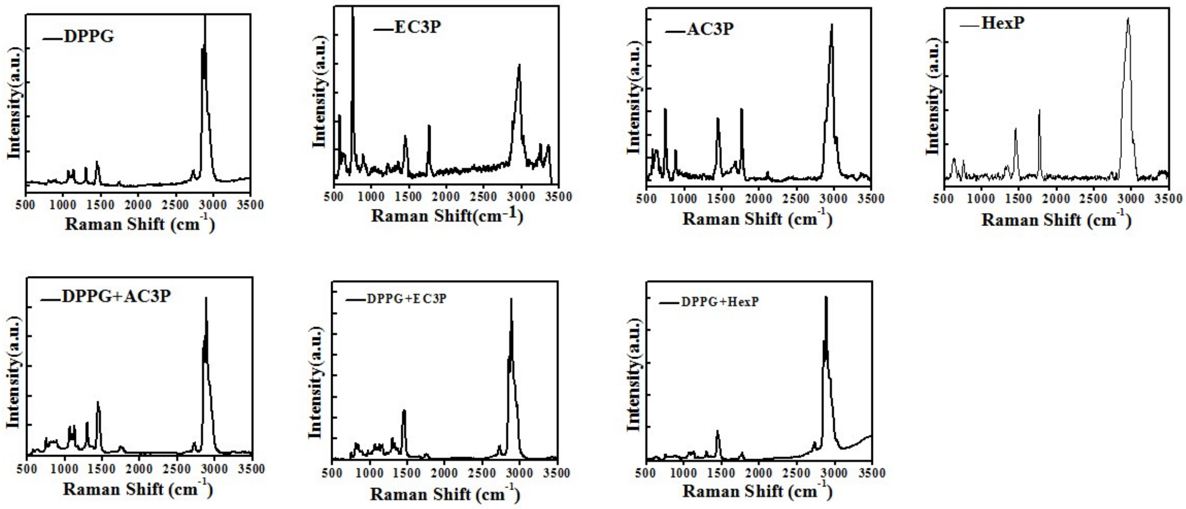

Figure S9. Full region Raman spectra of DPPG alone, polymer alone and DPPG + polymer.

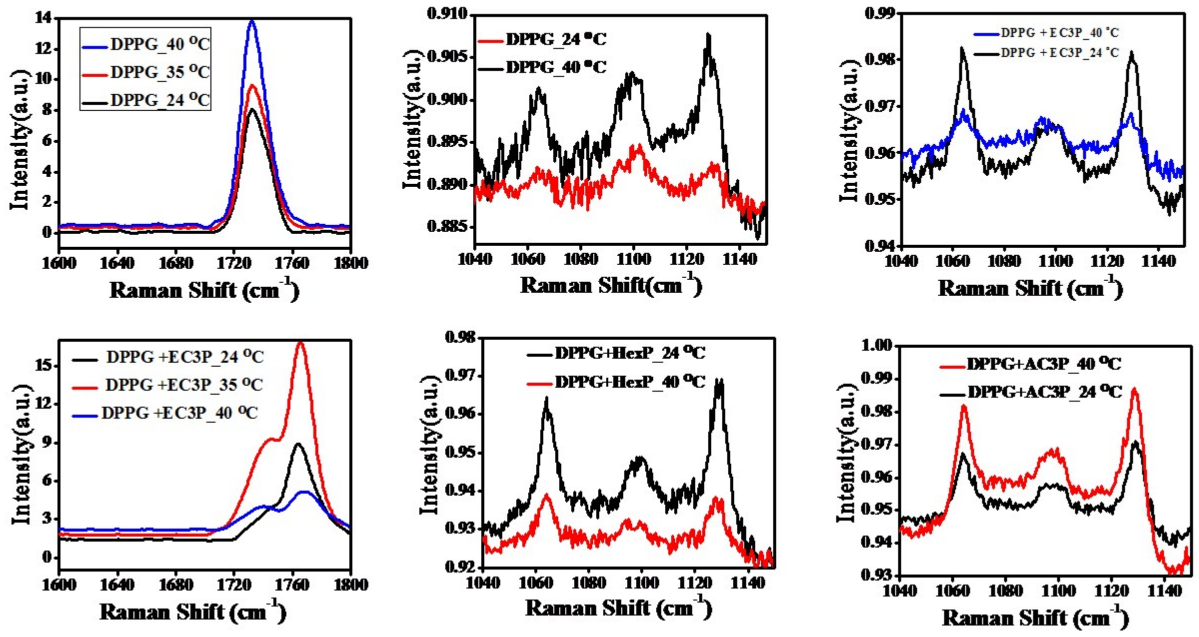

Figure S10. Temperature dependent Raman spectra of DPPG alone and DPPG + polymer.

Table S3. Parameters used for MD simulations.

System	N(polymers)	N(POPE)/ Leaflet	N(POPG)/ Leaflet	N(atoms)	Simulation time (ns)
Control	0	90	38	62945	300
Bilayer-AC3P	4	90	38	86138	150
Bilayer-EC3P	4	90	38	87672	150

Synthesis and Characterization

Synthesis of amide and ester based alkylating agents

***N-alkyl-1-bromoethanamide*:** Alkylamine (118 mmol) was dissolved in dichloromethane (55 mL). Potassium carbonate, K_2CO_3 (24.55 g, 178 mmol) was dissolved in 60 mL of distilled water and the solution was added to the organic solution. The resulting two phase solution was cooled to 4 °C. A solution of bromoacetyl bromide (35.85 g, 178 mmol) in dichloromethane (55 mL) was carefully added drop wise to the cooled solution while maintaining the temperature at 4 °C for about 30 min. Then the reaction mixture was stirred at room temperature for 12 h. The aqueous solution was separated and washed with dichloromethane (2 × 25 mL). The organic solution was washed with water (2 × 50 mL) and passed over the anhydrous Na_2SO_4 and concentrated to yield a white solid quantitatively.

***N-propyl-1-bromoethanamide*:** FT-IR: 3250 cm^{-1} (amide N-H str.), 2950-2850 (C-H str.), 1680 cm^{-1} (Amide I, C=O str.), 1560 cm^{-1} (Amide II, N-H ben.), 1470-1410 cm^{-1} (C-C str.), 1290-1110 (C-O str.); ^{1}HNMR (400 MHz, $CDCl_3$): δ/ppm 0.878 (t, terminal –CH_3, 3H), 1.543 (m, -CONHCH_2*CH_2*CH_3-, 2H), 3.278 (t, -CONH*CH_2*-, 2H), 3.881 (s, -CO*CH_2*Br, 2H), 6.475 (br s, amide –*NH*CO, 1H)); ^{13}C NMR (100 MHz, $CDCl_3$): δ 14.195, 22.768, 26.904, 29.324, 29.423, 29.588, 29.646, 29.708, 31.995, 40.403, 165.589; HR-MS: *m/z* 180.00 (observed): 179.99 (calculated for $[M+H]^+$).

Alkyl-1-bromoethanoate: Alcohol (116.5 mmol) was dissolved in dichloromethane (55 mL). Potassium carbonate, K_2CO_3 (19.32 g, 140 mmol) was dissolved in 60 mL of distilled water and the solution was added to the organic solution. The resulting two phase solution was cooled to 4 °C. A solution of bromoacetyl bromide (28.21 g, 140 mmol) in dichloromethane (55 mL) was carefully added drop wise to the cooled solution while maintaining the temperature at 4 °C for

about 30 min. Then the reaction mixture was stirred at room temperature for 12 h. The aqueous solution was separated and washed with dichloromethane (2 × 25 mL). The organic solution was washed with water (2 × 50 mL) and passed over the anhydrous Na_2SO_4 and concentrated to yield an oily liquid quantitatively.

Propyl-1-bromoethanoate: FT-IR: 2950-2850 (C-H str.), 1735 cm^{-1} (C=O str.), 1470-1410cm^{-1} (C-C str.), 1290-1110 (C-O str.); ^{1}HNMR (400 MHz, $CDCl_3$): δ/ppm 0.85 (t, terminal –CH_3, 3H), 1.57 (m, -$COOCH_2CH_2CH_3$, 2H), 4.0 (t, -$COOCH_2$-, 2H), 3.7 (s, -$COCH_2Br$, 2H); ^{13}C NMR (100 MHz, $CDCl_3$): δ 14.195, 22.768, 26.904, 29.324, 29.423, 29.588, 29.646, 29.708, 31.995, 40.403, 171.19; HR-MS: *m/z* 180.10 (observed): 179.98 (calculated for M^+).

Synthesis and characterization of polymeric derivatives

Poly(isobutylene-alt-N-(N',N'-dimethylaminopropyl)-maleimide) (PIBMI)

To a solution of 10 g of poly(isobutylene-*alt*-maleic anhydride) (PIBMA) (Avg. Mw = 6000 g/mol) in 60 mL of DMF, 7.96 g of 3-aminopropyldimethylamine (1.2 equivalents with respect to the monomer weight of the polymer (154 g/mol)) was added and stirred at 120 °C for 48 h in a screw-top pressure tube. The reaction mixture was cooled, precipitated with 200 mL of distilled water and was centrifuged at 10,000 rpm for 15 min. The polymer was dried at 55 °C for 24 h under vacuum to give a pale yellow solid with 100% yield (complete conversion of the anhydride to imide was confirmed by complete disappearance of peaks at 1850 cm^{-1} (C=O asym. str.) and 1785 (C=O sym. str.) for the anhydride ring and appearance of peaks 1767 cm^{-1} (C=O asym. str.), 1696 cm^{-1} (C=O sym. str.) for the imide ring by FT-IR).

PIBMI: FT-IR: 2950-2850 (C-H str.), 1767 cm^{-1} (C=O asym. str.), 1696 cm^{-1} (C=O sym. str.), 1470-1410cm^{-1} (C-C str.), 1290-1110 (C-O str.); ^{1}HNMR (400 MHz, $CDCl_3$): δ/ppm 0.7–1.2 (br $CH_2C\mathit{(CH_3)_2}$, 6H), 1.7 (br $\mathit{CH_2}C(CH_3)_2$, 2H), 1.86 (br $NCH_2\mathit{CH_2}CH_2N(CH_3)_2$, 2H), 2.2-2.5 (br $NCH_2CH_2\mathit{CH_2}N\mathit{(CH_3)_2}$, 8H), 2.7–3.1 (br, *CHCH*, 2H), 3.6 (br $N\mathit{CH_2}CH_2CH_2N(CH_3)_2$, 2H); ^{13}C NMR (100 MHz, $CDCl_3$): 179.9, 179.7, 179.4, 177.4, 177.3, 177.2, 55.5, 45.9, 45.5, 44.1, 40.8, 40.6, 40.2, 40.0, 37.4, 26.2, 25.5, 24.8, 24.7, and 24.6.

Synthesis of polymeric quaternized derivatives

To a solution of 0.5 g of PIBMI in 20 mL of dry DMF/dry $CHCl_3$ (1:1), 2 equivalents (with respect to the monomer weight of PIBMI) of alkyl-1-bromoethanoate or *N*-alkyl-1-bromoethanamide was added and stirred at 65 °C (for ester) or 75 °C (for amide) for 96 h in a screw top pressure tube. The solution was cooled, precipitated with 40 mL of diethylether and filtered. The white solid was washed with diethylether (4 × 40 mL) and dried at 40 °C for 6 h under vacuum. The percentage of conversion given by the degree of quaternization was calculated from ^{1}H-NMR and was found to be in the range of 90-95 % for all the derivatives.

EC3P: FT-IR: 2950-2850 (C-H str.), 1767 cm^{-1} (imide C=O asym. str.), 1696 cm^{-1} (imide C=O sym. str.), 1735 cm^{-1} (ester C=O str.) 1470-1410cm^{-1} (C-C str.), 1290-1110 (C-O str.); ^{1}HNMR (400 MHz, D_2O): δ/ppm 0.85 (br, terminal –$\mathit{CH_3}$, 3H), 0.95–1.2 (br, $CH_2C\mathit{(CH_3)_2}$, 6H), 1.57 (br,-$COOCH_2\mathit{CH_2}CH_3$, 2H), 1.7 (br, $\mathit{CH_2}C(CH_3)_2$, 2H), 2.0 (br, $NCH_2\mathit{CH_2}CH_2N(CH_3)_2$, 2H), 2.7–3.1 (br, *CHCH*, 2H), 3.1-3.3 (br, $NCH_2CH_2\mathit{CH_2}N\mathit{(CH_3)_2}$, 8H), 3.6 (br, $N\mathit{CH_2}CH_2CH_2N(CH_3)_2$, 2H), 3.7 (br, -$N(CH_3)_2\mathit{CH_2}CO$, 2H) 4.0 (br, -$COO\mathit{CH_2}$-, 2H).

AC3P: FT-IR: 3250 cm^{-1} (amide N-H str.), 2950-2850 (C-H str.), 1767 cm^{-1} (imide C=O asym. str.), 1696 cm^{-1} (imide C=O sym. str.) 1680 cm^{-1} (amide I, C=O str.), 1560 cm^{-1} (Amide II, N-H

ben.), 1470-1410cm^{-1} (C-C str.), 1290-1110 (C-O str.); ^{1}HNMR (400 MHz, D_2O): δ/ppm 0.878 (br, terminal –CH_3, 3H), 0.95–1.2 (br, $CH_2C(CH_3)_2$, 6H), 1.543 (br, -$CONHCH_2CH_2CH_3$, 2H), 1.7 (br, $CH_2C(CH_3)_2$, 2H), 2.0 (br, $NCH_2CH_2CH_2N(CH_3)_2$, 2H), 2.7–3.1 (br, *CHCH*, 2H), 3.1-3.3 (br, $NCH_2CH_2CH_2N(CH_3)_2$, 8H), 3.5 (br, -$CONHCH_2$-, 2H), 3.6 (br, $NCH_2CH_2CH_2N(CH_3)_2$, 2H), 3.8 (br, -$N(CH_3)_2CH_2CO$, 2H).
